# The Role of T Cell Immunity in Monoclonal Gammopathy and Multiple Myeloma: From Immunopathogenesis to Novel Therapeutic Approaches

**DOI:** 10.3390/ijms23095242

**Published:** 2022-05-08

**Authors:** Ivana Lagreca, Giovanni Riva, Vincenzo Nasillo, Patrizia Barozzi, Ilaria Castelli, Sabrina Basso, Francesca Bettelli, Davide Giusti, Angela Cuoghi, Paola Bresciani, Andrea Messerotti, Andrea Gilioli, Valeria Pioli, Corrado Colasante, Daniela Vallerini, Ambra Paolini, Monica Maccaferri, Francesca Donatelli, Fabio Forghieri, Monica Morselli, Elisabetta Colaci, Giovanna Leonardi, Roberto Marasca, Leonardo Potenza, Rossella Manfredini, Enrico Tagliafico, Tommaso Trenti, Patrizia Comoli, Mario Luppi

**Affiliations:** 1 Section of Hematology, Department of Surgical and Medical Sciences, University of Modena and Reggio Emilia, AOU Modena, 41124 Modena, Italy; patrizia.barozzi@unimore.it (P.B.); ilaria.castelli@unimore.it (I.C.); francesca.bettelli@unimore.it (F.B.); davide.giusti@unimore.it (D.G.); cuoghi.angela@policlinico.mo.it (A.C.); bresciani.paola@aou.mo.it (P.B.); messerotti.andrea@aou.mo.it (A.M.); gilioli.andrea@aou.mo.it (A.G.); pioli.valeria@aou.mo.it (V.P.); corrado.colasante@unimore.it (C.C.); daniela.vallerini@unimore.it (D.V.); paolini.ambra@aou.mo.it (A.P.); maccaferri.monica@policlinico.mo.it (M.M.); f.donatelli@piafondazionepanico.it (F.D.); fabio.forghieri@unimore.it (F.F.); morselli.monica@policlinico.mo.it (M.M.); colaci.elisabetta@aou.mo.it (E.C.); leonardi.giovanna@policlinico.mo.it (G.L.); roberto.marasca@unimore.it (R.M.); leonardo.potenza@unimore.it (L.P.); 2 Diagnostic Hematology and Clinical Genomics, Department of Laboratory Medicine and Pathology, AUSL/AOU Modena, 41124 Modena, Italy; g.riva@ausl.mo.it (G.R.); vincenzo.nasillo@unimore.it (V.N.); enrico.tagliafico@unimore.it (E.T.); t.trenti@ausl.mo.it (T.T.); 3 Pediatric Hematology/Oncology Unit and Cell Factory, Istituto di Ricovero e Cura a Carattere Scientifico (IRCCS) Policlinico San Matteo, 27100 Pavia, Italy; s.basso@smatteo.pv.it (S.B.); pcomoli@smatteo.pv.it (P.C.); 4 Centre for Regenerative Medicine “S. Ferrari”, University of Modena and Reggio Emilia, 41125 Modena, Italy; rossella.manfredini@unimore.it

**Keywords:** MGUS, multiple myeloma, plasma cells, T cell immunity, tumor microenvironment, immunotherapy

## Abstract

Multiple Myeloma (MM) is a malignant growth of clonal plasma cells, typically arising from asymptomatic precursor conditions, namely monoclonal gammopathy of undetermined significance (MGUS) and smoldering MM (SMM). Profound immunological dysfunctions and cytokine deregulation are known to characterize the evolution of the disease, allowing immune escape and proliferation of neoplastic plasma cells. In the past decades, several studies have shown that the immune system can recognize MGUS and MM clonal cells, suggesting that anti-myeloma T cell immunity could be harnessed for therapeutic purposes. In line with this notion, chimeric antigen receptor T cell (CAR-T) therapy is emerging as a novel treatment in MM, especially in the relapsed/refractory disease setting. In this review, we focus on the pivotal contribution of T cell impairment in the immunopathogenesis of plasma cell dyscrasias and, in particular, in the disease progression from MGUS to SMM and MM, highlighting the potentials of T cell-based immunotherapeutic approaches in these settings.

## 1. Introduction

Multiple Myeloma (MM) is a hematologic malignancy characterized by the accumulation of clonal plasma cells (PCs) within the bone marrow (BM), typically producing a monoclonal immunoglobulin (M-protein), readily detectable in blood and/or urine samples. The MM diagnosis classically requires both the demonstration of clonal BM plasma cells ≥ 10% and the presence of end-organ damage and/or myeloma-defining events (MDE) [1]. MM represents about 1% of all cancers and 10% of all hematological malignancies, being the second most common blood cancer after non-Hodgkin’s lymphoma and chronic lymphocytic leukemia (CLL) [1].

In the last 40 years, the treatment for MM has significantly evolved, progressively improving patients’ survival rates. Since the 1980s, the first advance came with the clinical development of autologous stem cell transplant (SCT) [2]. In the late 1990s, the introduction of proteasome inhibitors (PIs) and immunomodulatory drugs (IMiDs) made a big impact on the therapeutic strategy for MM [3]. Recently, monoclonal antibodies, such as daratumumab, elotuzumab, isatuximab, belantamab mafodotin, as well as histone deacetylating agents, such as panobinostat, selinexor and other investigational agents [4,5,6] have successfully extended the therapeutic options for MM patients [7,8]. In addition, chimeric antigen receptor T cell (CAR-T) therapy is now emerging as a promising approach to treat relapsed/refractory MM (RRMM) [9]. Ancillary care for myeloma-related bone disease and other interventions have also resulted in improved survival rates [5,6,10]. However, despite such great therapeutic progress, MM largely remains an incurable disease, with the majority of patients experiencing recurrent relapses, with increasingly shorter periods of remission until death, due to disease-related organ failure and treatment-related complications [1].

MM is classically preceded by a pre-neoplastic state, called monoclonal gammopathy of undetermined significance (MGUS), which typically carries a rate of progression to MM of 1% per year. MGUS is defined by serum M-protein of <3 g/dL, clonal BM plasma cells < 10%, and the absence of end-organ damage or MDEs [11]. Some patients may show a type of intermediate disease stage between MGUS and MM, termed smoldering multiple myeloma (SMM). By definition, SMM is an asymptomatic malignant condition, characterized by higher levels of M-protein and/or of clonal BM plasma cells compared to MGUS, while sharing with MGUS the absence of end-organ damage and MDEs, and is associated with a 10% annual risk of evolution to overt MM [1,5,6,12,13].

The MGUS-to-MM progression is a complex, multistep immunobiological phenomenon, characterized by the acquisition of both genetic abnormalities in the clonal PC population and immune modifications in the BM microenvironment. The initiating events seem to occur in the germinal center, possibly during the error-prone process of rearranging somatic DNA in B cells by V(D)J recombination, class-switch recombination, and somatic hypermutation. Common primary genetic events include hyperdiploidy and translocations of the IgH locus, which are usually mutually exclusive. Late oncogenic events, such as secondary translocations, copy-number variants, oncogenic mutations, and epigenetic alterations drive the progression of the malignant PC population [14]. Major genetic events occur early during disease evolution and are already found in patients with premalignant stages of the disease, suggesting that they are necessary, but not sufficient for MM pathogenesis [15]. Consistent with this, the whole genome analysis of unique paired samples from SMM patients progressing to MM revealed that the genomic landscape at the smoldering stage, including mutational profile and structural rearrangements, is remarkably similar to MM [16]. Moreover, the mutational burden of MGUS/SMM patients who did not progress to MM was found to be equivalent to the mutational burden of progressors [17], thus further suggesting that additional non-genomic alterations are required for disease progression.

As a matter of fact, it is now well recognized that changes in the BM microenvironment contribute to MGUS-to-MM evolution, providing a “dis-inflammatory” niche able to promote tumor growth, and, in particular, facilitating immune evasion through local immune suppression and inhibition of antitumor effector lymphocytes [18]. In line with this immunopathogenetic view—immune dysfunction is a key mechanism of MGUS-to-MM disease progression—strong evidence exists that malignant PCs can be specifically recognized and killed by cytotoxic T cells. Here, we review the important contributions of the immune system in either controlling or promoting myeloma outgrowth, briefly describing MM-associated abnormalities in BM cell composition and immunosurveillance, focusing on the impairments of specific T cell functions. Finally, we discuss the emergence of novel immunotherapeutic approaches, aimed to trigger the T cell-mediated elimination of myeloma cells.

## 2. Myeloma-Promoting Immunological Changes of the BM Microenvironment Contribute to MGUS-to-MM Progression

**Figure 1:**
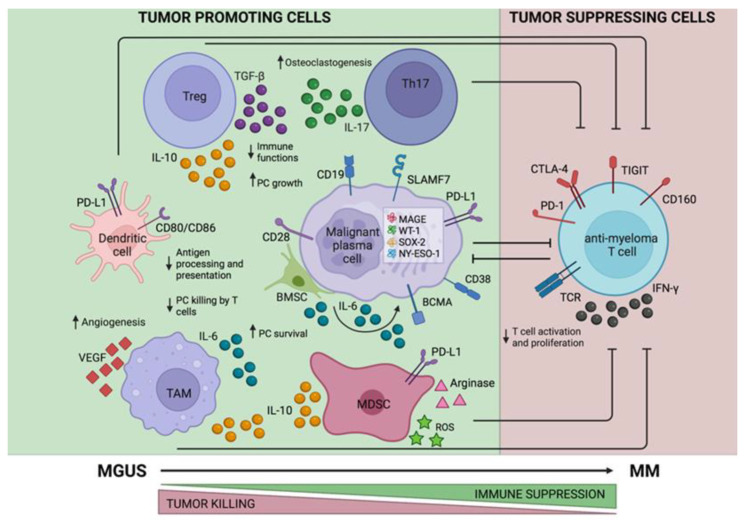
MM-associated immunological BM niche. Disease progression from MGUS to MM is associated with changes in the BM immune microenvironment, with progressive impairment of *tumor suppressing cells* (mainly anti-MM effector T cells) and accumulation of *tumor promoting cells*, such as regulatory T cells (Tregs), Th17 cells, tumor associated macrophages (TAMs), myeloid-derived suppressor cells (MDSCs), and suppressive dendritic cells (DCs). ↓: decrease; ↑: increase; ⊥: inhibition; PC: plasma cell; BMSC: bone marrow stromal cells; SLAMF7: signaling lymphocyte activation molecule family 7; BCMA: B-cell maturation antigen; MAGE: melanoma-associated antigen; WT1: Wilms’ tumor 1; SOX-2: SRY-box transcription factor 2; NY-ESO-1: New York esophageal squamous cell carcinoma 1; PD-L1: programmed death ligand 1; IL-6: interleukin 6; IL-10: interleukin 10; IL-17: interleukin 17; TGF-β: transforming growth factor β; VEGF: vascular endothelial growth factor; ROS: reactive oxygen species; IFN-γ: interferon γ; TCR: T cell receptor; PD-1: programmed cell death protein 1; CTLA-4: cytotoxic T-lymphocyte antigen 4; TIGIT: T cell immunoreceptor with Ig and ITIM domains.

Long-lived plasma cells primarily reside in the BM niche, and both normal and malignant PCs establish complex and reciprocal interactions with the cellular components, extracellular matrix (ECM) proteins, and soluble factors that are essential for their survival [18]. Along with different genomic mutations progressively occurring in plasma cells during MGUS-to-MM progression [14], the immune remodeling of the BM microenvironment also plays a fundamental role in such disease evolution, facilitating cancer cell survival and proliferation, development of drug resistance, and failure of the antineoplastic immune response (Figure 1). Multiple cellular components can act in a redundant and compensatory manner to sustain malignant PC outgrowth. In this context, bone marrow mesenchymal stem cells (MSCs) are important players, promoting PC homing in the BM through the secretion of CXCL12 (the ligand of CXCR4), providing contact-dependent support to PCs by integrins and secreting pro-survival, anti-apoptotic, and pro-angiogenic cytokines, such as interleukin 6 (IL-6), vascular endothelial growth factor (VEGF), and insulin-like growth factor 1 (IGF-1) [19]. Furthermore, MM-associated MSCs can induce the secretion of pro-osteoclastogenic factors, including pro-inflammatory cytokines and RANKL (receptor activator of nuclear factor –k ligand), inducing osteoclast differentiation and suppression of osteoblasts, resulting in osteolytic bone disease [20].

Moreover, different types of immune cells are known to represent pivotal regulators of the BM microenvironment in the progression toward MM. The immune composition of the MM-associated microenvironment is substantially different from that observed in healthy individuals. The elevated BM concentrations of IL-6 and transforming growth factor-β (TGF-β) determine increased numbers of Th17 cells in MM patients, compared to MGUS patients and healthy donors, and this, in turn, favors a general immunosuppressive state [21,22]. Indeed, Th17 subsets are able to suppress cancer immunosurveillance by secreting IL-17 and IL-10. IL-17 can also activate osteoclastogenesis, contributing to MM bone disease [23].

In parallel, the expansion of (i) regulatory T cells (Treg), (ii) tumor-associated macrophages (TAMs), and (iii) myeloid-derived suppressor cells (MDSCs) help to maintain such immunosuppressive milieu. In particular, Tregs are able to inhibit cytotoxic T cells and the specific functions of antigen presenting cells (APC) via direct cell-to-cell contact and by releasing IL-10 and TGF-β [24]. Furthermore, Tregs are increased in the peripheral blood (PB) of MM and MGUS patients, compared to healthy controls [25], and higher frequencies of functional Treg correlate with a worse prognosis [26,27]. Of note, Treg/Th17 balance in MM patients, compared to MGUS stage, is remarkably skewed toward Tregs, becoming a signature of the immunosuppressive state [21]. Interestingly, the immunological profile associated with long-term survival of MM includes a recovery of the Treg/Th17 balance [28].

TAMs are generally characterized by M2-like phenotype, showing limited phagocytic activity with low production of pro-inflammatory cytokines, as well as poor antigen-presenting capability, resulting in the inhibition of both T cells and natural killer (NK) cells. In line with this, MM-associated TAMs are a remarkable source of IL-6 and IL-10, thus promoting tumor growth while fostering immune suppression [29]. Moreover, MM-associated macrophages are able to stimulate angiogenesis through the release of VEGF-α and nitric oxide (NO), and also to differentiate into endothelial cell-like cells under the autocrine/paracrine effect of VEGF-α [30]. MDSCs are immature myeloid precursors showing broad immunosuppressive functions, which are induced by an abnormal BM cytokine milieu in chronic inflammatory conditions, such as cancer, infections, and trauma [31,32]. This immature cell subset exerts a direct antiproliferative effect against lymphocytes via increased NO production, L-arginine depletion, and IL-10 secretion [33]. In addition, MDSCs indirectly inhibit effector T cell responses by inducing immunosuppressive Tregs [34].

Furthermore, the aberrant function of dendritic cells (DCs) has also been described in the MM-associated BM microenvironment [35], showing their ability to directly favor myeloma cell growth and survival. [36] Of note, the accumulation of DCs has been reported in the BM of MM patients, where they promote tolerance to tumor antigens and T cell evasion via interaction of CD80/CD86 with CD28 expressed in the tumor cells [37]. DCs are also known to activate Tregs, as well as Th17 cells, in MM [38,39] and to express high levels of PD-L1, thus importantly contributing to immune dysfunctions and T cell exhaustion [36,40].

Interestingly, when effective anti-MM therapies are used, the altered immune BM niche of MM patients may undergo a significant reversal of immunosuppressive features, possibly moving back to the pre-neoplastic immunological state of MGUS [18]. Some detailed effects of single immunomodulatory treatments on the tumor microenvironment are reported in the last section of the review. Indirectly, this general observation further supports the idea that a dis-immune process in the BM microenvironment is pivotal for MGUS-to-MM progression. However, whether such therapy-induced immunological modifications of different immune cell types in the BM may be implemented as relevant prognostic factors in the clinical management of MM patients still requires further investigations.

## 3. Progressive Impairment of Effector Immune Functions in MGUS and MM Patients

Several numerical, phenotypic, and functional abnormalities in the T cell repertoire have been demonstrated in the clinical course of MM patients. Quantitatively, a decrease in the CD4+/CD8+ ratio, due to a reduction in the total number of CD4+ T cells, with a relative increase in CD8+ T cells, is observed during disease progression [41]. Beyond such quantitative changes, MM patients show a relevant impairment of effector T cell functions. Under the effect of TGF-β, released by regulatory immune cells, as well as by MM cells, T cells present a notable reduction in IL-2-mediated autocrine proliferation [42]. Moreover, multiple signaling defects, such as the downregulation of CD28, CD152, CD3-zeta chain, p56lck, ZAP-70, and PI3K, are also observed in CD4+ and CD8+ T cells, especially in patients with advanced-stage MM [43].

During disease progression, the T cell phenotype undergoes notable changes. Different T cell subsets, with a stem-like profile and a tissue-resident signature, are lost during MGUS-to-MM transition [44]. Moreover, patients with advanced MM show the emergence of senescent and exhausted T cells. In particular, MM-associated CD8+ T cells were demonstrated to upregulate several inhibitory receptors related to exhaustion, such as PD-1, CTLA-4, 2B4, and CD160. Consistent with these findings, such T lymphocytes progressively displayed an immunological signature of senescence, with expression of CD57 and a lack of CD28. In addition, these T cells exhibited a lower proliferative capacity, impaired cytotoxic function, and an inability to produce IFN-γ after antigenic stimulation [45]. Interestingly, this senescent/exhausted phenotype was found to be telomere-independent, with elevated telomerase activity and normal-for-age telomere lengths [46]. Coupled with the overexpression of PD-L1 on MM plasma cells, the increased expression of PD-1 in cytotoxic T cells is now considered a fundamental mechanism of immune tolerance in MM. Of note, the expression of PD-L1 is higher in plasma cells from MM patients compared to MGUS patients and healthy subjects, and PD-L1 expression in malignant PCs is associated with an increased risk of progression from SMM to symptomatic MM. PD-L1 upregulation has also been described in RRMM patients, compared to newly diagnosed cases (NDMM), and MM patients with detectable residual disease after treatment showed higher levels of PD-L1 compared with the results at diagnosis [47]. These findings have promoted clinical studies investigating the therapeutic inhibition of the PD-1/PD-L1 pathway in MM patients. However, monotherapy with PD-1/PD-L1 inhibitors has yielded unsatisfactory results, and even the combination of anti-PD-1 with IMiDs, such as lenalidomide or pomalidomide, showed limited clinical benefits in MM patients [48,49,50]. Recently, a new immune checkpoint called TIGIT (T cell immunoreceptor with immunoglobulins and ITIM domain) is gaining interest as a potential target for MM therapy. TIGIT is expressed at a higher level in BM CD8+ T cells from NDMM or RRMM patients, compared to healthy donors, and it represents the most common inhibitory molecule found in MM-associated CD8+ T cells. Importantly, the TIGIT blockade can restore the effector function of CD8+ T cells in MM patients and protect mice from MM development when used as single agent [51], as well as in combination with SCT [52].

In addition, the oligoclonal expansions of CD57+ CD8+ terminal effector T cells (T_TE_ cells) have been observed in both the BM and PB of MM patients [53,54], but their pathogenetic role and clinical significance are still controversial. Two opposite interpretations have been proposed: one suggests that oligoclonal T_TE_ cells represent exhausted and senescent effector T cells and, thus, their expansion should represent a detrimental feature for anti-myeloma T cell immunity [45]; in the other view, by considering that oligoclonal T_TE_ cells can readily emerge in MM patients under thalidomide treatment, and their presence is associated with improved survival [55], these cells could rather be relevant for the immune control of aberrant PCs proliferations. However, to date, similar investigations on oligoclonal T_TE_-cell expansions following treatments with lenalidomide, pomalidomide, and new-generation IMIDs (e.g., iberomide), have not yet been reported in the literature. Such oligoclonal expansions of T_TE_ in MM patients may result from persistent stimulation of CD8+ T cells in the BM in the absence of effective clearance of the malignant clone. Furthermore, these T_TE_ cells in the BM can be grouped on the basis of CD69 expression [54], a marker of tissue residence [56]. CD69− T_TE_ cells circulate between the PB and BM, while CD69+ T_TE_ cells are restricted to the BM and share many features in common with T resident memory (T_RM_) cells [57]. Among healthy controls, both CD69− and CD69+ T_TE_ cells were found in comparable proportions, while CD69− cells are predominant in MGUS and SMM. On the other hand, NDMM predominantly showed either CD69− or CD69+ cells [54]. The CD69− T_TE_ cells showed oligoclonal expansion and the ability to lyse autologous tumor cells, while CD69+ T_TE_ cells exhibited low perforin and granzyme expression and increased immune checkpoint expression. Thus, the balance between CD69− T_TE_ and CD69+ T_TE_ cells may likely regulate anti-myeloma responses and contribute to clinical heterogeneity in MM patients [54].

Alongside the T cell impairment, NK cells were also found to be functionally defective in MM patients. Although increased NK-cell frequencies were reported in both MGUS and MM patients, NK cells in MGUS patients still preserve their cytotoxic functions, while progression to MM is associated with the decline in NK-cell cytotoxicity, mainly mediated by the downregulation of the NK activating receptors, including NKG2D. In a complementary manner, high levels of MHC class I on MM cells can efficiently provide inhibitory signaling mediated by killer immunoglobulin-like receptor (KIR) on NK cells. Moreover, NK cells in MM patients have increased PD-1 expression, which results in the suppression of NK cytotoxic activity by PD-L1-expressing MM cells [58].

## 4. Evidence of Myeloma-Specific T Cell Responses in MGUS and MM Patients

Different findings have highlighted the importance of an active myeloma-specific immunosurveillance in the control of aberrant PC proliferation. Several research groups have reported substantial evidence of a remarkable T cell recognition and immune activation in the MM setting. As the first identified myeloma-specific antigen, the variable region of the secreted monoclonal immunoglobulin (idiotope, Id) was found to stimulate naturally occurring specific T cell responses in MGUS and MM patients. Moreover, Id-specific cytotoxic T cell lines were generated ex vivo and showed the ability to kill autologous MM cells in a specific manner [59,60,61].

To date, several tumor-associated antigens, such as WT1, SOX-2, RHAMM, PRAME, and a series of cancer/testis antigens (namely, MAGE-A1/A2/A3/A6, MAGE-C1/C2, NY-ESO-1, and Melan-A/MART-1), have also been discovered to be expressed by malignant PCs and to induce detectable T cell responses, often with remarkable killing capacity against neoplastic PCs in vitro [62,63,64,65,66,67,68,69,70,71,72,73,74,75].

Originally, nearly 20 years ago, it was demonstrated that effective anti-myeloma CD8+ T cells—able to exert cytolytic and IFN-γ-producing responses toward autologous malignant PCs—can be generated from the PB and BM of MM patients after ex vivo stimulation with DCs pulsed with autologous tumor cell lysates, while freshly isolated T cells were not reactive against autologous MM cells [63]. Conversely, freshly isolated CD4+ and CD8+ BM T cells from MGUS patients were readily able to display vigorous responses against autologous BM PCs, thus strongly suggesting that anti-myeloma T cell immunity is lost during the progression from MGUS to MM [64]. This impairment is probably due to T cell exhaustion caused by the increasing tumor burden and chronic exposure to target MM antigens [45]. Interestingly, T cells isolated from MGUS or MM patients recognized autologous—but not allogeneic—tumor cells, indicating that anti-myeloma T cell responses are clone-specific and patient-specific [63,64]. Other studies reported high frequencies of CD4+ and CD8+ T cells directed against some cancer/testis antigens or SOX-2 in MGUS patients, showing significant associations with a reduced risk of progression to MM, improved disease control, and longer survival [65,66,67,68]. Furthermore, NY-ESO-1-specific T cells from MM patients were shown to specifically kill primary MM cells [69]. In addition, other antigens highly expressed by malignant PCs have been identified as targets of specific anti-myeloma T cell responses. Dickkopf-1 (DKK1) is a secreted protein that specifically inhibits Wnt/β-catenin signaling and thus, contributes to osteolytic bone disease in MM. Immunogenic HLA-A2-restricted DKK1-derived peptides were identified and DKK1 peptide-specific CD8+ T cells were detected, although at low frequencies, in a series of MM patients. DKK1 peptide-specific cytotoxic T lymphocytes (CTLs) able to lyse DKK1-expressing cells (including autologous primary MM cells) were generated from healthy blood donors and MM patients, after stimulation by autologous DCs loaded with DKK1 peptides [76].

XBP1 (X-box binding protein 1), CD138 (syndecan-1), and CS1 (SLAMF7) are important antigens associated with MM pathogenesis and are typically expressed on malignant PCs. Immunogenic peptides derived from these target antigens were identified and their ability to generate antigen-specific CTLs was reported [77,78]. Of note, such peptides are able to elicit highly effective anti-myeloma T cell immunity in SMM patients [79]. Moreover, recent in silico analysis in MM samples allowed for the identification of some further immunogenic mutation-derived neoantigens, able to induce specific T cell activation, which was associated with antitumor activity in vitro and clinical response in vivo [80]. The main MM-specific T cell responses described in the literature so far are summarized in Table 1.

Furthermore, in the setting of SCT and donor lymphocyte infusions (DLIs), the efficacy of such donor immunity-based therapeutic approaches classically resides in the so-called *graft-versus-myeloma* effect, thus confirming de facto that the anti-myeloma immunity is fundamental for disease control in MM patients [81,82,83,84]. Intriguingly, the emergence of WT1-specific T cell responses after allogeneic SCT have been reported in a series of MM patients, demonstrating a clear-cut correlation between the occurrence of WT1-specific CTLs and the therapeutic response, particularly in patients treated with DLIs [70]. As a matter of fact, this is the first evidence of specific anti-myeloma T cells contributing to the *graft-versus-myeloma* effect [70]. However, there is a significant body of evidence also supporting a possible autologous *graft*-*versus*-*myeloma* effect. Early clinical studies have reported a direct correlation between lymphocyte count recovery after autologous SCT and improved overall survival in a wide range of diseases [85]. In MM patients, after autologous SCT, a high number of CD4+ T cells and an increased CD4+/CD8+ ratio were significantly associated with better outcomes [86]. More specifically, it was shown that the augmentation of autologous SCT with autologous lymphocyte infusions, combined with immunotherapeutic products containing recombinant MAGE-A3 protein, is able to generate a robust MAGE-A3-specific CD4+ T cell immune response, highlighting that the period around graft re-infusion provides a favorable milieu for additional immunotherapy, including tumor-antigen vaccination [71].

Of interest, anti-myeloma T cell immunity in the autologous transplant setting was further investigated by using the Vk*MYC murine myeloma model [87]. Myeloma-bearing mice receiving BM transplantation from myeloma-naive or myeloma-experienced donor mice showed a broad induction of T cell-dependent myeloma control, with improved survival and reduced myeloma progression. This effect was chiefly mediated by memory T cells within myeloma-experienced grafts, but also through the priming of naive T cells after engraftment. Such protective anti-myeloma immunity was clone-specific and mainly conferred by IFNγ-secreting CD8+ T cells, also being further enhanced by a CD137 agonist and IL-17A inhibition, thus representing a potential therapeutic approach to improve the autologous *graft-versus-myeloma* effect [87].

## 5. Therapeutic Strategies to Restore Specific T Cell Immunosurveillance against MM

Clonal progression of myeloma cells is supported by a permissive immunological microenvironment, typically characterized by loss of effective immunosurveillance, with pivotal impairment of protective tumor-specific T cell responses. In this view, novel immunotherapeutic approaches, aiming at providing a targeted stimulation of the immune system against neoplastic PCs, are emerging as a promising strategy to elicit deeper and more durable therapeutic responses in MM patients.

IMiDs (e.g., thalidomide, lenalidomide, and pomalidomide) display both myeloma “on-target” effects and favorable immunologic “off-target” effects [89]. They induce cereblon-dependent degradation of the transcription factors Ikaros (IKZF1) and Aiolos (IKZF3), leading to transcriptional repression of IRF4 and MYC and resulting in myeloma cell apoptosis and stimulation of T and NK cell activity [90,91]. Specifically, IMiDs boost T cell proliferation, enhance IL-2 and IFN-γ production, and reduce IL-10 secretion by CD4+ and CD8+ T cells, also inducing NK cell activation and proliferation, as well as antibody-dependent cellular cytotoxicity (ADCC) [89,92]. Moreover, IMiDs suppress Treg expansion in vitro [93], improve tumor antigen uptake by DCs, and boost the efficacy of the antigen presentation process [94]. In line with these anti-myeloma activities, such immunomodulatory drugs have contributed to significantly improve the outcome of MM patients and are currently the backbone of several MM treatment regimens, especially in combination with other emerging immunologic strategies. Indeed, in combination with emerging monoclonal antibodies (see below), IMiDs have shown significant synergistic effects, providing an improved overall response rate (ORR) and extension of both progression-free survival (PFS) and overall survival (OS) in MM patients [95,96,97]. In high-risk SMM patients, the immunomodulatory effects of lenalidomide, even when combined with low-dose dexamethasone, are able to reactivate the protective anti-myeloma immunity [98], thus contributing to the significant delay of the progression toward symptomatic disease [99,100,101].

As previously mentioned, myeloma cells can evade immunosurveillance through the upregulation of ligands of inhibitory immune receptors, thus inducing a functional exhaustion in protective T cells. Several studies suggested the importance of the immune checkpoint axis PD-1/PD-L1 in MM, but early clinical trials reported a lack of efficacy of anti-PD-1 nivolumab monotherapy [102]. Better responses to immune checkpoint inhibition have been demonstrated in combination with IMiDs [103], even if such approaches resulted in relevant toxicities [49,50]. Of note, in preclinical studies, anti-PD-1 monotherapy, when administered early after SCT, was highly effective against myeloma [52,87,104]. Moreover, post-SCT combination of anti-PD-1 blockade with IMiDs was reported to induce notable improvement in the complete response (CR) rate in high-risk MM patients [105], providing further evidence for the implementation of immunotherapy early after autologous SCT. TIGIT represents an additional inhibitory checkpoint, known to be overexpressed on T cells of MM patients, and to negatively regulate T cell functions [106]. Anti-TIGIT therapeutics are under clinical investigation in patients with RRMM, either alone (NCT04354246) or in combination (NCT04150965).

Significantly, advances in TAMs biology (related to tumor metabolism and immunity) have provided a strong rationale for testing novel immunotherapies specifically aimed to repolarize TAMs toward antineoplastic activities (M1-like TAMs) and revert the immunosuppressive tumor microenvironment [107,108]. Different experimental immunotherapeutic strategies for M2-to-M1 macrophage polarization have been described so far, including cytokine milieu modulation by a combination of GM-CSF and M2 cytokine inhibitors (e.g., anti-MIF) [109], microRNA inhibitors (e.g., anti-MiR-16) [110], and transcription factor inhibitors (e.g., anti-STAT3) [111]. Moreover, monoclonal antibodies (moAb) targeting myeloma surface antigens can elicit anti-MM activity through different mechanisms, including both a direct cytotoxic effect (via apoptosis) on MM cells, and immune-mediated cytotoxicity, such as ADCC, antibody-dependent cellular phagocytosis (ADCP), and complement-dependent cytotoxicity (CDC) [112]. Recently, US and European drug regulatory agencies have approved different antibodies targeting CD38 (daratumumab and isatuximab) or SLAMF7 (elotuzumab) for MM treatment [113]. Daratumumab is the first anti-CD38 antibody approved by the Food and Drug Administration (FDA) for previously-treated MM patients. Beyond the direct killing of CD38-expressing MM cells by ADCC and CDC, it also induces the eradication of CD38+ MDSCs, Tregs, and Bregs, promoting a restoration of antitumor immune responses associated with NK and T cell activation and oligoclonal expansions [114]. As a single agent, daratumumab demonstrated rapid, deep, and durable responses in RRMM patients [115,116,117]. Furthermore, daratumumab in combination with novel agents, remarkably improved the outcomes of both RRMM and NDMM patients [95,118,119,120]. More recently, isatuximab, a new CD38-directed moAb, has been approved, in combination with IMiDs, to treat RRMM patients [97]. Finally, elotuzumab is a moAb specifically directed against SLAMF7, a glycoprotein expressed on myeloma cells, able to promote the killing of myeloma cells, mainly through NK-mediated ADCC, as well as by preventing interactions between myeloma cells and BM stromal cells [121,122,123,124].

In addition, moAb-based technologies have also provided the framework for the development of antibody-drug conjugates (ADCs) and bispecific T cell engagers (BiTEs). The former consists of a monoclonal antibody targeting a myeloma-specific surface antigen which is directly linked to a cytotoxic drug (e.g., chemotherapy). After binding its target, ADCs release the chemotherapeutic agent, resulting in myeloma cell death, with limited damage to healthy cells and reduced side-effects, contextually providing strong immune-mediated cytotoxicity [125]. Currently, several ADCs are under clinical development for MM treatment. Among these drugs, belantamab mafodotin, an anti-B-cell maturation antigen (BCMA) ADC, has shown impressive therapeutic activity as a single agent in a phase II clinical trial of RRMM patients, leading to its approval as a monotherapy by both the FDA and the European Medicine Agency (EMA) [126,127]. BiTEs are engineered molecules able to simultaneously bind tumor-specific antigens and T cells, mediating functional T cell activation and the killing of tumor cells [128]. Importantly, they work irrespective of the MHC haplotype and co-stimulation and do not require peptide antigen presentation [129,130]. By considering some encouraging pre-clinical results, several clinical trials have started testing bi-specific agents directed against MM-associated antigens, such as BCMA, CD38, CD19, GPRC5D, and FcRH5 [131]. In particular, the most clinically advanced BiTE therapy in the MM setting, namely AMG 701, is targeted against BCMA. This drug has been tested in a cohort of heavily-pretreated MM patients, providing an overall response rate (ORR) of 70%, including 50% minimal residual disease (MRD)-negative complete responses at the maximum tolerated dose [132]. Nonetheless, the approval of these compounds is hampered by several barriers, mainly related to treatment-associated toxicities (specifically cytokine release syndrome, CRS) or to the emergence of tumor immune evasion (i.e., BCMA downregulation) [133,134]. Thus, this approach still needs to be optimized for a safer and more effective application. In particular, it should be considered that BiTE efficacy requires the presence of functional T cell responses, making this therapy more attractive after SCT or in NDMM patients.

An additional strategy to boost MM-specific immunity is represented by cellular therapies, including either adoptive T cell (ACT) or engineered CAR-T cell approaches. Importantly, CAR-T therapy has emerged as a revolutionary treatment for patients with B-cell malignancies, leading to the approval of two anti-CD19 products: tisagenlecleucel and axicabtagene ciloleucel [135,136]. CAR-T cells are genetically modified T lymphocytes expressing a specific receptor able to recognize and bind the antigen of interest on target cells, independently from MHC haplotype and antigen presenting machinery [137]. By considering the selective expression of BCMA on PCs (both normal and neoplastic) [138], several anti-BCMA CAR-T products are currently tested in clinical trials for heavily pre-treated RRMM patients, reporting ORRs of 64-88% with remarkably deep responses [139,140,141]. Other CAR-T cells targeting CD138, SLAMF7 and GPRC5D have recently entered in early stages of clinical testing, after the encouraging preclinical results [142,143,144]. However, CAR-T therapy is often associated with specific, albeit manageable, adverse events, such as CRS and neurotoxicity [145]. Moreover, most patients eventually relapse, probably because of some CAR-T cell extrinsic factors, such as the loss of target antigen and immunosuppressive action of the BM tumor microenvironment [138]. Another postulated mechanism of disease progression is the lack of long-term persistence of CAR-T cells. Several strategies are being developed to increase the proportion of long-lived CAR-T cells with a memory phenotype in the infused product, which is expected to result in longer CAR-T persistence [146,147,148]. In addition, the time lag between the collection and manufacturing of autologous CAR-T cells remains a challenge for patients with progressive disease. Allogeneic CAR-T cells from healthy donors can provide readily available “off the shelf” CAR-T products. Allogeneic products targeting BCMA and SLAMF7 are currently under clinical development [149,150].

However, despite the potential benefits, allogeneic CAR-T cells can cause graft-versus-host disease (GvHD), which is associated with relevant morbidity and mortality. Secondly, a possible lack of persistence through rapid elimination by the host immune system is another main issue of these products [151,152]. To overcome CAR-T cell safety concerns, alternative killer immune cell subsets are currently being explored as CAR vessels. NK cells represent an appealing candidate population, as they are highly cytotoxic, but are not associated with GvHD, thereby showing higher potential for allogenic manufacturing [153,154]. A further ideal CAR vector for allogeneic therapy is represented by invariant natural killer T (iNKT) cells. The iNKT cells recognize glycolipid antigens presented by highly conserved CD1d via their defined and invariant T cell receptor. Due to this specific antigen recognition pattern, iNKT cells are not associated with GvHD, and can even prevent it. Moreover, iNKT cells themselves show strong anti-tumor effects in tumor models via the CD1d-mediated killing of CD1d-positive tumor cells and immunosuppressive TAMs and MDSCs [155,156]. Of note, malignant plasma cells have been reported to highly express CD1d, and, in line with this notion, both CD38- and BCMA-CAR iNKT cells were able to effectively eliminate MM cells [156].

Along with the BCMA-targeting CAR-T cell approaches, other novel strategies of ACT with BCMA-targeting CTLs are also under investigation. To date, immunogenic HLA-A2-specific BCMA-derived peptides allowed the ex vivo generation of highly functional BCMA-specific CD8+ CTLs, with effective killing of myeloma cells, characterized by IFN-γ, TNF-α and, IL-2 production [88]. Hence, such BCMA-derived peptide pool could be exploited for therapeutic applications in MM patients, either by vaccination approaches or as a specific stimulation for ex vivo expansion of anti-myeloma CTLs. During the in vitro expansion phase, by using immune agonists (e.g., OX40), checkpoint inhibitors (e.g., anti-LAG-3 moAb), or nanoparticle-based delivery systems, BCMA-specific CTLs can acquire better immune functions [88,157]. In addition, an interesting clinical study has demonstrated the feasibility and efficacy of the autologous use of marrow-infiltrating lymphocytes (MILs), able to generate a persistent myeloma-specific T cell immunity, which was associated with PFS increase [158].

## 6. Perspectives and Conclusions

MGUS-to-MM evolution is characterized by the subtle emergence of an aberrant PC clone acquiring multiple genetic lesions, within a subverted BM microenvironment, which progressively becomes more permissive to the neoplastic proliferation. Among immunological changes promoting myeloma outgrowth, the loss of protective effector functions and, in particular, the impairment of myeloma-specific T cell immunosurveillance, play a key role. Consistent with this, the restoration of a functional anti-myeloma T cell immunity can represent an effective treatment option for advanced disease (as evidenced by CAR-T therapy in RRMM patients), and in turn, some immunological approaches could even be investigated at earlier stages (i.e., MGUS and SMM) to prevent the progression to symptomatic MM. In this view, the identification of novel specific T cell markers and the validation of significant T cell profiles may provide valuable new prognostic tools in the management of both MGUS and MM patients, possibly integrating with the emerging use of MRD, as assessed by next-generation sequencing and flow cytometry (NGS and NGF) [159]. In perspective, the prognostic monitoring of either spontaneous or therapy-induced anti-myeloma T cell responses, readily detectable in the PB or BM of MGUS/MM patients, may reasonably help guide the individualized use of adoptive T cell treatments in these settings. Additional investigations are warranted to further integrate the growing knowledge on myeloma immunopathogenesis, novel immunological biomarkers, and recent advanced in T cell-based immunotherapies.

## Figures and Tables

**Table 1:** Summary of main studies describing tumor-specific T cell responses in MM and MGUS patients.

References	Target Antigens	Disease Setting (Total Patients)	Sample Source	Immunoassays	Main Results
Spontaneous T Cell Responses					
**Dhodapkar et al., 2003** [64]	Whole tumor/preneoplastic cells	MM (12) MGUS (6)	PB, BM	ELISPOT, ICS	T cell responses to autologous premalignant plasma cells were detected in the BM of patients with MGUS, while tumor-specific T cell effector functions were absent in the BM of MM patients.
**Van Rhee et al., 2005** [69]	NY-ESO-1	MM (3)	PB	ICS, Tetramer analysis, ^51^Cr-release-assay	Spontaneous NY-ESO-1-specific T cells were found in PB of MM patients, and were able to kill primary MM cells.
**Spisek et al., 2007** [65]	SOX-2	MM (14) SMM (21) MGUS (16)	PB	Luminex, ICS	Spontaneous T cell responses against SOX2 were detected in MGUS patients, but not in MM patients.
**Goodyear et al., 2008** [66]	MAGE-A1/A2/A3	MM (53 + 32) MGUS (25 + 30)	PB	IFN-γ CSA, ^51^Cr release assay	CD4+ T cell immunity to MAGE proteins was stronger and more frequent in MGUS, compared with MM.
**Tyler et al., 2013** [70]	WT-1	MM (24)	PB, BM	ICS, Tetramer analyses	WT1-specific CTLs incremented after allogeneic T cell-depleted SCT + DLI and elicited a graft-versus-myeloma effect.
**Dhodapkar et al., 2015** [68]	SOX-2	SMM (155) MGUS (132)	PB	Luminex	Anti-SOX2 T cells were detected in PB from MGUS and SMM patients, and correlated with reduced risk of progression to symptomatic MM.
**Cohen et al., 2019** [71]	MAGE-A3	MM (13)	PB	ELISPOT, ICS	Autologous lymphocyte infusion associated with MAGE-A3 vaccination elicited antigen-specific T cell immunity in autologous SCT patients.
**Perumal et al., 2020** [80]	Mutation-derived neoantigens	MM (184)	PB	ICS, CFSE-based cytotoxicity assay	Shared neoantigens were detected across MM patients and were able to induce specific T cell activation associated with in vitro antitumor activity and clinical responses.
**Ex vivo Generated** **T-Cell Responses**					
**Dhodapkar et al., 2002** [63]	Whole tumor cells	MM (7)	PB, BM	ELISPOT, ^51^Cr release assay	In vitro stimulation with DCs loaded with autologous tumor cells generated tumor-specific cytolytic T cell responses.
**Qian et al., 2007** [76]	DKK1	MM (n.a.)	PB	Proliferation assay, ^51^Cr-release-assay, ELISPOT	DKK1-specific CTLs were generated from PB of MM patients and efficiently lysed DKK1-expressing cells, including primary myeloma cells.
**Christensen et al. 2009** [72]	Melan-A/MART-1	MM (n.a.)	PB	ELISPOT, ^51^Cr-release-assay	Ex vivo expanded Melan-A-specific T cells were able to lyse autologous MM cells.
**Greiner et al., 2010 [73]; Schmitt et al., 2008 [74]**	RHAMM	MM (7)	PB	ELISA, ELISPOT, Tetramer analysis, ^51^Cr-release-assay	Peptide vaccination with RHAMM-derived peptide R3 induced specific CD8+ effector T cells and positive clinical effects.
**Racanelli et al., 2010** [67]	Plasma cell lysates, NY-ESO-1	MM (20) MGUS (20)	BM	^51^Cr-release-assay	In vitro expanded antitumor CD8+ T cells in the BM of MM patients showed a reduced cytotoxic potential, compared with MGUS patients.
**Ocadlikova et al., 2010** [75]	hTERT, MUC-1	Healthy subjects (n.a.)	PB	CSA; flowcytometric cytotoxicity test	DCs loaded with hTERT- and MUC1-derived peptides were able to generate specific CTLs with anti-myeloma cytotoxic activity.
**Bae et al.,** **2015** [79]	XBP-1, CD138, CS1 (SLAMF7)	SMM (8)	PB	Proliferation assay, ICS, CD107a degranulation	Multipeptide-specific CTLs were generated from SMM patients’ T cells and showed effective anti-MM responses.
**Bae et al.,** **2019** [88]	BCMA	Healthy subjects (n.a.)	PB	Proliferation assay, ICS, CD107a degranulation	BCMA-derived peptides were able to induce specific CTLs, showing polifunctional Th1-specific immune activities against MM.

NY-ESO-1: New York esophageal squamous cell carcinoma 1; SOX-2: SRY-box transcription factor 2; MAGE-A1/A2/A3: melanoma-associated antigen 1/2/3; WT1: Wilms’ tumor 1; DKK1: Dickkopf-1; Melan-A/MART1: melanoma antigen recognized by T cells; RHAMM: receptor for hyaluronan mediated motility; hTERT: human telomerase reverse transcriptase; MUC1: mucin 1; XBP1: X-box binding protein 1; CD138: syndecan-1; CS1 (SLAMF7): signaling lymphocyte activation molecule family 7; BCMA: B-cell maturation antigen; PB: peripheral blood; BM: bone marrow; ELISA: enzyme linked immunosorbent assay; ELISPOT: enzyme-linked immunoSPOT; ICS: intracellular cytokine staining; CSA: cytokine secretion assay; ^51^Cr: ^51^Chromium; CFSE: carboxyfluorescein succinimidyl ester;; SCT: stem cell transplant; CTLs: cytotoxic T lymphocytes; MM: Multiple Myeloma; SMM: Smoldering Multiple Myeloma; MGUS: Monoclonal Gammopathy of Undetermined Significance.
